# Voriconazole therapeutic drug monitoring including analysis of CYP2C19 phenotype in immunocompromised pediatric patients with invasive fungal infections

**DOI:** 10.1007/s00228-024-03752-z

**Published:** 2024-09-06

**Authors:** Matylda Resztak, Paulina Zalewska, Jacek Wachowiak, Agnieszka Sobkowiak-Sobierajska, Franciszek K. Główka

**Affiliations:** 1 Department of Physical Pharmacy and Pharmacokinetics, Poznan University of Medical Sciences, Poznań, Poland; 2 Department of Pediatric Oncology, Hematology and Transplantology, Poznan University of Medical Sciences, Poznań, Poland

**Keywords:** Voriconazole, Trough concentration, Genetic polymorphisms, Therapeutic drug monitoring, Children

## Abstract

**Purpose:**

Therapeutic drug monitoring (TDM) of voriconazole (VCZ) should be mandatory for all pediatric patients with invasive fungal infections (IFIs). The narrow therapeutic index, inter-individual variability in VCZ pharmacokinetics, and genetic polymorphisms cause achieving therapeutic concentration during therapy to be challenging in this population.

**Methods:**

The study included 44 children suffering from IFIs treated with VCZ. Trough concentrations (C_trough_) of VCZ ware determined by the HPLC-FLD method. Identification of the CYP2C19*2 and CYP2C19*17 genetic polymorphisms was performed by PCR–RFLP. The correlation between polymorphisms and VCZ C_trough_ was analyzed. Moreover, the effect of factors such as dose, age, sex, route of administration, and drug interactions was investigated.

**Results:**

VCZ was administered orally and intravenously at a median maintenance dosage of 14.7 mg/kg/day for a median of 10 days. The VCZ C_trough_ was highly variable and ranged from 0.1 to 6.8 mg/L. Only 45% of children reached the therapeutic range. There was no significant association between C_trough_ and dosage, age, sex, route of administration, and concomitant medications. The frequencies of variant phenotype normal (NM), intermediate (IM), rapid (RM) and ultrarapid metabolizers (UM) were 41%, 18%, 28%, and 13%, respectively. C_trough_ of VCZ were significantly higher in NM and IM groups compared with RM, and UM groups.

**Conclusion:**

The C_trough_ of VCZ is characterized by inter-individual variability and a low rate of patients reaching the therapeutic range. The significant association exists in children between VCZ C_trough_ and CYPC19 phenotype. The combination of repeated TDM and genotyping is necessary to ensure effective treatment.

## Introduction

Invasive fungal infections (IFIs) are life-threatening diseases in immunocompromised patients, including those undergoing oncological treatment and/or allogeneic hematopoietic stem cell transplantation (allo-HSCT), immunosuppressive treatment or suffering from primary immunodeficiencies [1–3]. Voriconazole (VCZ) is a second-generation triazole agent with potent spectrum antifungal activity against *Aspergillus* species, *Candida* species, and molds that are other triazoles resistant [4–6] and is recommended as a first choice for the treatment of IFIs.However, incomplete response to therapy and toxicity can cause ineffective treatment of IFIs [7–9].The efficacy of VCZ depends on the drug’s minimal inhibitory concentration (MIC) against the pathogen. A preclinical model of disseminated *Candida albicans* infection has shown that the area under the curve (AUC)/MIC ratio is the best predictor of VCZ efficacy [8, 10–12]. Trough concentrations (C_trough_) are a practical alternative in pharmacokinetics studies of VCZ [12, 13].Numerous observational studies have recommended a range of VCZ C_trough_ of 1- 5.5 mg/L as adequate for treatment [14–17], while C_trough_ of < 1 mg/L was associated with increased mortality [18–22].In children, VCZ dosing has been particularly challenging; drug plasma concentrations are unpredictable, and the target range of C_trough_ is often not reached, even if recommended doses are administered. Several factors, including nonlinear pharmacokinetics, the narrow therapeutic index, age, liver function, drug-drug interaction, and variable oral bioavailability, particularly in children, are responsible for large inter- and intra-patient variability of VCZ pharmacokinetics [23–28].

VCZ is metabolized by the cytochrome P450 enzyme system, specifically by isozymes CYP2C19, CYP2C9, and CYP3A. A growing number of studies have documented that CYP2C19 polymorphism makes a crucial contribution to the extensive pharmacokinetic variability of VCZ [22, 29]. CYP2C19 phenotype variants are known to be strongly correlated with differences in the therapeutic plasma concentration of VCZ [30, 31]. Genetic polymorphism of CYP2C19 is associated with 30% to 50% variation in VCZ metabolism between individuals [32]. The impact of CYP2C19 genetic variants on VCZ metabolism and variability in exposure in adults has been acknowledged. However, data available in the pediatric group are limited [33–36]. Most of the TDM VCZ studies have been carried out on a small population of children and it has been challenging to evaluate statistically significant correlations [30, 37, 38]. Therefore, whether the genotype-directed dossing of VCZ allows for optimized therapy in pediatric patients needs more studies.

In this paper, we aimed to describe the results obtained from TDM in pediatric patients treated in our children’s hospital and to investigate the contribution of genetic polymorphism in CYP2C19 to the variability in C_trough_ of VCZ. A better understanding of the impact of these polymorphisms on VCZ exposure may help achieve individualized drug dosing and ultimately improve the clinical outcome.

## Methods

### Study population

All procedures performed in the studies involving human participants were in accordance with the ethical standards of the institutional and national research committee and with the 1964 Helsinki Declaration and its later amendments or comparable ethical standards. The study was approved by the Bioethical Committee at Poznan University of Medical Sciences with the ethics code of 1106/12, 535/18, and 299/18. Informed consent was obtained from the parents or guardians prior to initiating the study.

The study included 44 pediatric patients (31 male and 13 female) of Caucasian origin aged 2 to 17 years (median age 12.5 years) treated in a single children’s university hospital over a 9-year period (from 2013 to 2021). The median body weight was 38.8 kg (range 14–89 kg). The inclusion criteria were diagnosis of the invasive fungal infection (IFI) and treatment with at least 5 days of VCZ therapy. Studied children were stratified into two groups according to age, i.e. < 12 years (n = 18) and ≥ 12 years (n = 26), to determine if there was equal representation of younger and older patients among the covariates. The demographic and clinical characteristics of patients included are summarized in Table 1. There were no significant differences among the two age groups in any of the covariates. The most frequent underlying condition was hematological malignancy (16; 36%). Fifteen children (34%) received allogeneic hematopoietic stem cell transplantation (HSCT) before using VCZ.

**Table 1:** Children's demographic and clinical characteristics

**Parameter**	**Total N = 44**	** < 12 years N = 18 (41%)**	** ≥ 12 years N = 26 (59%)**
Age, years (median, range)	12.5 (2–17)	7.5 (2–11)	14 (12–17)
Sex, male [ n (%)]	31 (70.4)	31 (70.4)	20 (70.4)
Body weight, kg (median, range)	38.8 (14–89)	24.7 (14–47)	39.3 (27–89)
Underlying conditions			
Hematopoietic stem cell transplant	15 (34)	7 (47)	8 (53)
Hematologic malignancies	16 (36)	6 (38)	10 (62)
Other	13 (30)	6 (33)	7 (27)
Route of administration [ n (%)]			
Intravenous	11 (25)	4 (9)	7 (16)
Oral	33 (75)	14 (32)	19 (43)
Median VCZ dose, mg/kg/day (range)	14.7 (4.5–28.6)	17.4 (8.0–28.6)	8.3 (4.5–18.6)
Median duration of VCZ treatment, days (range)	10 (5–48)	10 (5–48)	9 (5–39)
VCZ C_trough_ (mg/L) (median, range)	0.8 (0.1–6.8)	1.2 (0.1–4.4)	0.7 (0.1–6.8)
< 1 mg/L, [n (%)]	23 (55)	10 (55)	15 (58)
1–5.5 mg/L, [n (%)]	20 (43)	8 (44)	10 (38)
> 5.5 mg/L, [n (%)]	1 (2)	0 (2)	1(4)
Concomitant medications			
PPIs	9 (20)	1(6)	8 (31)
CsA	15 (34)	7 (47)	8 (53)
ALB (g/dL)	3.8 (2.7–5.8)	3.9 (2.7–4.9)	3.8 (3.1–5.8)
C_cr_ (mg/dL)	0.4 (0.2–1.2)	0.3 (0.2–0.9)	0.5 (0.2–1.2)
ALT (U/L)	30 (7–218)	25 (7–218)	31 (11–130)
AST (U/L)	23.5 (6–95)	24 (12–89)	23.5 (6–95)

*PPIs*-proton pump inhibitors, *CsA*–cyclosporine, *ALB*-serum albumin level, *ALT*-alanine transaminase, *AST*-aspartate aminotransferase

### VCZ therapy

The dosing of VCZ administration for all patients was decided by the clinicians according to the Summary of Product Characteristics (Table 2) and current European Conference on Infections in Leukemia (ECIL) recommendations [39]. During the therapy, in some cases, dose adjustments were performed by the pediatrician based on treatment response. The administration route of VCZ was oral or intravenous infusion, depending on the condition of the patient. The medical record of each patient was reviewed using a data collection template. The following data were considered: age, sex, body weight, IFI diagnosis, treatment dose, treatment time duration, administration routes, and factors that could potentially influence VCZ C_trough_ such as level of serum albumin (ALB), alanine transaminase (ALT), aspartate aminotransferase (AST) as well as concomitant medications.

**Table 2:** Current VCZ dosing recommendations from the summary of product characteristics [39]

Patient age/weight	Recommended dose by treatment type			
	Intravenous		Oral	
	Loading	Maintenance	Loading	Maintenance
> 2–12 years or 12–14 years/ < 50 kg	9 mg/kg/12 h	8 mg/kg/12 h		9 mg/kg/12 h (max 350 mg/12 h)
> 14 years / < 50 kg	6 mg/kg/12 h	4 mg/kg/12 h	200 mg/12 h	100 mg/12 h
> 12–14 years/ > 50 kg	6 mg/kg/12 h	4 mg/kg/12 h	400 mg/12 h	200 mg/12 h

### Blood sampling and analytical assays

All drug concentrations were drawn as part of routine care, with the decision to perform TDM based on the care team. Approximately 1 ml of full blood samples were collected at a steady state just before administration of the next VCZ dose. Plasma was collected and centrifuged at 1620 g for 10 min and stored at -20 °C until analyzed. Trough plasma concentration (C_trough_) of VCZ was measured at least 5 days after the VCZ initiation. VCZ C_trough_ was determined using a previously published HPLC method with fluorescence detection elaborated and validated in our laboratory [40]. Separation conditions were the following: LiChrospher RP-18e column (125 mm × 4 mm; 5 μm); mobile phase consisted of acetonitrile: potassium dihydrogen phosphate buffer adjusted to pH 6.5 at 35:65 (v/v); flow rate of 1.2 ml/min; excitation and emission wavelength were 254 nm and 385 nm; respectively; column temperature 30 °C and sample size inject of 50 μL. Intra and interday precision were within 2.2 to 13.4% and 0.4 to 1.4%; respectively. The linearity range was 0.05 -10.0 mg/L.

### Diagnostic criteria and treatment outcome

Patients were diagnosed with fungal infection according to the definitions of the European Organization and Treatment of Cancer/Mycoses Study Group (EORTC/MSG). Therapy response was assessed during hospitalization using clinical, radiological, and microbiological criteria. Treatment success was evaluated based on partial or complete improvement in clinical radiological and microbiological signs of infection. Treatment failure was defined as persistent or progressing IFI.

### CYP2C19 genotyping

Blood samples (3 ml EDTA) for genotype detection were collected from each patient. Of the 44 blood samples collected, 39 were tested for the *CYP2C19* phenotype in association with blood hemolysis in five samples. The isolation of the genomic DNA from 200 μL of whole blood was performed with a commercially available kit according to the instructions supplied by the manufacturer (GenMATRIX Quick Blood Purification Kit, EURx, Poland).

Two single nucleotide polymorphisms that define the major CYP2C19 alleles—rs4244285, G > A (*2 allele) and rs12248560, C > T (*17 allele) were carried out by polymerase chain reaction-restriction fragments length polymorphism (PCR–RFLP) using a technique previously described [41]. Genotypes were identified based on the analysis of the restriction fragment lengths. Endonucleases used for restriction and lengths of restriction fragments are presented in Table 3.

**Table 3:** Conditions of PCR–RFLP reaction for studied genotypes

**Polymorphism**	**Primers**	**Annealing temperature °C**	**Length of PCR product**	**Restriction enzyme**	**Length of restriction fragments**
CYP2C19*2	F: 5’-CAGAGCTTGGCATATTGTATC-3’ R: 5’- GTAAACACAAAACTAGTCAATG-3’	59	321 bp	SmaI	*1/*1, 212 bp, 109 bp *1/*2, 321 bp, 212 bp, 109 bp *2/*2, 321 bp
CYP2C19*17	F: 5’-TCTGGGGCTGTTTTCCTT-3’ R: 5’-CACCTTTACCATTTAACCCCCTA-3’	56	375 bp	SfaNI	*1/*1, 188 bp, 152 bp, 35 bp *1/*17, 223 bp, 188 bp, 152 bp, 35 bp *17/*17, 223 bp, 152 bp

*F*-forward primer, *R*–reverse primer, *bp*–base pairs

Confirmation of the CYP2C19 polymorphisms was performed by the sequencing technique using 3130 × 1 Genetic Analyzer (Applied Biosystems HITACHI, USA). The results were analyzed using the Sequence Scanner v1.0 and MegAlign v5.05 computer program.

Patients were classified into categories of metabolizer phenotypes based on Clinical Pharmacogenetics Implementation Consortium (CPIC) guidelines [42, 43]. According to this system, patients expressing the CYP2C19*1*2 genotype were defined as intermediate metabolizers (IM), those expressing CYP2C19*1*17 as rapid metabolizers (RM), and those with CYP2C19*17*17 as ultrarapid metabolizers (UM). The normal metabolizers (NM) were assigned by default to patients without a *2 or *17 allele.

### Statistical analysis

All statistical tests were performed using STATISTICA 13.0 software (StatSoft, Inc., Tulsa, OK, USA). A *p*-value lower than 0.05 was considered significant. All of the data were non-normally distributed, therefore the results are presented as median and range.

Shapiro–Wilk test was performed to establish the normality of the data. For non-normally distributed data, Spearman correlation analysis, Mann–Whitney test, and Kruskal–Wallis test were used. Multivariate analysis by linear regression was performed to estimate the determinants contributing to the variability of VCZ C_trough_ including age, sex, presence of the studied alleles, liver function, and treatment with proton pump inhibitor (PPI), and cyclosporine (CsA).

## Results

VCZ was administered orally to 75% of the patients (33/44) and 25% (11/44) of the patients received intravenous doses. The median maintenance dose of VZC was 14.7 (range 4.5–28.6) mg/kg/day and the median duration of VCZ treatment was 10 (range, 5–48) days. Concomitant medications noted with potential drug-drug interactions with VCZ were PPIs (n = 9) and CsA (n = 15). Due to this small sample size, no statistically significant effect was observed of concomitant drugs on the C_trough_ of VCZ.. The median levels of ALB, ALT, AST, and creatinine concentration were in the normal reference value range (Table 1). Although in individual cases it was clearly above the range.

### Analysis of VCZ plasma concentration

**Fig. 1:**
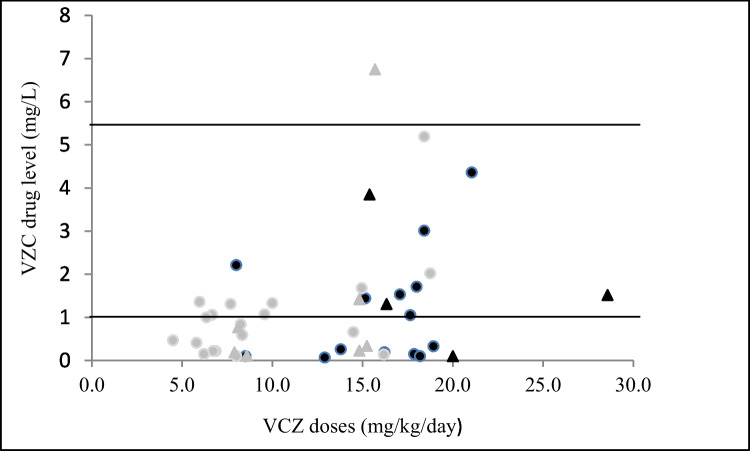
VCZ C_trough_ level by weight-adjusted dosage received. The black and gray circles represent data after receiving oral VCZ in patients aged < 12 years and ≥ 12 years, respectively. The black and gray triangles represent data after receiving intravenous VCZ in patients aged < 12 years and ≥ 12 years, respectively. The area between the horizontal lines indicates the therapeutic range 1.0–5.5 mg/L

**Fig. 2:**
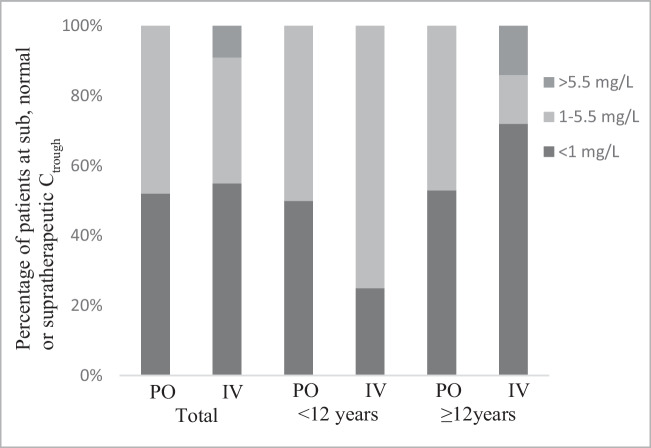
VCZ C_trough_ distribution (%) per formulation per age group; C_trough_: trough concentration; PO: oral administration, IV: intravenous administration

A total of 44 VCZ C_trough_s were measured in this research. The results of TDM in patients are shown in Table 1. The median of the VCZ C_trough_ values was 0.8 (range, 0.1–6.8) mg/L. Forty-three percent of children (19/44) achieved an adequate VCZ level. Fifty-seven percent of patients (25/44) were out of the therapeutic range. From them, 55% (21/44) and 2% (1/44) were in subtherapeutic (< 1 mg/L) and supratherapeutic (> 5.5 mg/L) concentrations, respectively. Figure 1 shows the distribution of measured VCZ C_trough_ and their respective doses. No significant differences in the number of VCZ C_trough_ within the therapeutic range between patients < 12 years and ≥ 12 years (P = 0.192) were observed. No predictable relationship between dose and VCZ C_trough_ was found in analyzed patients. (P = 0.267). No significant difference was investigated in median VCZ C_trough_ between children who were treated with oral and intravenous administration in both groups (P = 0.903 and P = 0.71) (Fig. 2). Additionally, in the current study, no significant effect of sex on VCZ C_trough_ was observed.

### Response to VCZ therapy

Outcome data were analyzed in patients treated with antifungals. Of the 44 patients, 31 (70%) were considered treatment successes and 13 (30%) were treatment failures. Among patients who successfully completed treatment, 20 (64%) had VCZ levels equal to or above 1.0 mg/l, compared with 1 (8%) among patients who failed treatment. A relationship was found between C_trough_ values and response to treatment. The median VCZ C_trough_ was significantly lower in patients who failed treatment 0.19 mg/L (range: 0.1–1.68 mg/L) than in patients treated successfully 1.31 mg/L (range: 0.10–6.75 mg/L) (P < 0.05).

### Genotype analysis

**Fig. 3:**
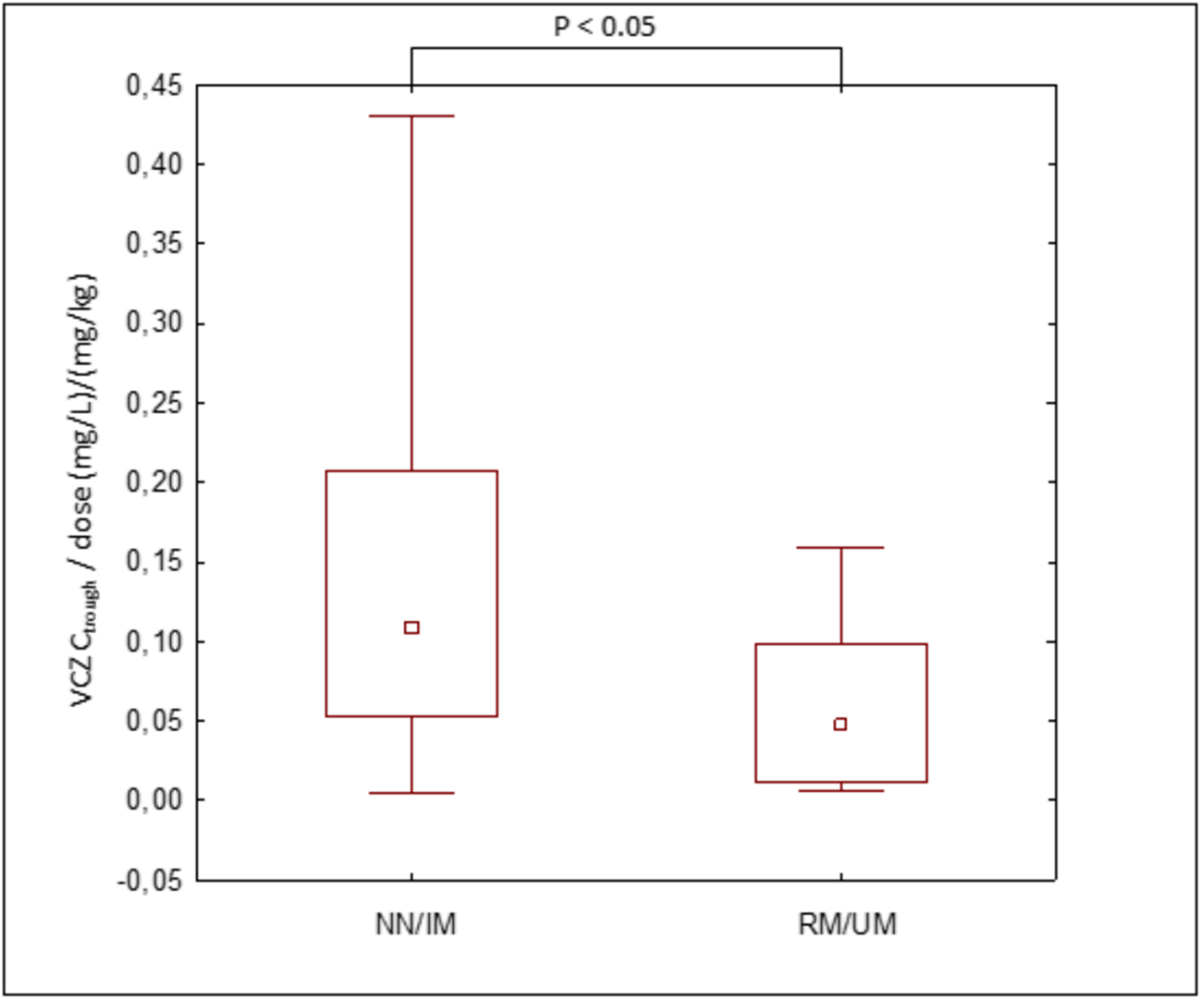
Influence of *CYP2C19* phenotype on dose-normalized VCZ C_trough_. Box plots represent the medium, 25th, and 75th percentiles, P < 0.05 was considered to indicate statistical significance

Depending on the determined *CYP2C19* phenotype, the subjects were divided into four groups: normal metabolizer NM; intermediate metabolizer IM; rapid metabolizer RM, and ultrarapid metabolizer UM*.* No poor metabolizers PM were found in our patient’s population. The numbers and frequencies of variants of alleles are displayed in Table 4. The wild-type allele CYP2C19*1*1 was observed most frequently (41%) followed by the CYP2C19*1*17 allele (28%), CYP2C19*1*2 allele (18%), and CYP2C19*17*17 allele (13%). The differences of VCZ C_trough_ among the *CYP2C19* phenotypes are presented in Table 4. The VCZ C_trough_ within the therapeutic range in CYP2C19*1*1 and CYP2C19*1*2 genotypes were observed with a median of value 1.32 (range, 0.10–6.75) mg/L, and 1.42 (range, 0.22–4.36) mg/L, respectively. The VCZ C_trough_ in children with the CYP2C19*1*17 and CYP2C19*17*17 genotypes were observed. Medians of VCZ C_trough_ were under the target concentration of 1.0–5.5 mg/L and amounted 0.23 (range, 0.10–1.31) mg/L, and 0.41 (range, 0.15–1.53) mg/L, respectively. In the total group of CYP2C19, only 51% reached the therapeutic level of VCZ. A subtherapeutic C_trough_ was observed in 46% of cases, whereas the C_trough_ was above 5.5 mg/l in only one case (3%). More patients with versus without the RM;UM/NM;IM phenotype had a subtherapeutic C_trough_ (64%; 80% vs. 31%; 29%, respectively), Table 5. Patients homozygous and heterozygous CYP2C19*17 had lower dose-normalized VCZ C_trough_ than those with a CYP2C19*2 dyplotypes (median 0.024; range 0.024–0.159 mg/L/mg/kg and 0.071; range 0.008–0.102 mg/L/mg/kg, respectively), Table 4. A similar relationship was observed in groups of children < 12 and ≥ 12 years of age. For the age group < 12 years the medians level ratio of VCZ C_trough_ to daily dose in the CYP2C19*1*17, CYP2C19*1*1, CYP2C19*1*1, and CYP2C19*1*2 dyplotypes were 0.010; range 0.010–0.080 mg/L/mg/kg, 0.017; range 0.017–0.089 mg/L/mg/kg, 0.077; range 0.005–0.250 mg/L/mg/kg, and 0.242; range 0.207–0.276 mg/L/mg/kg, respectively. However, in the group ≥ 12 years, the median for the mentioned dyplotypes were 0.064; range 0.009–0.159 mg/L/mg/kg, 0.086; range 0.070–0.102 mg/L/mg/kg, 0.123; range 0.013–0.430 mg/L/mg/kg, and 0.096; range 0.033–0.228 mg/L/mg/kg, respectively, Table 4. The relationship between individual C_trough_ corrected to the daily VCZ dose and *CYP2C19* diplotypes is presented in Fig. 3. The significant difference was observed in the C_trough_ of VCZ between the CYP2C19 phenotypes (P < 0.05).

**Table 4:** Relationship between CYP2C19 phenotype and VCZ exposure

**CYP2C19 dyplotype**	**N (%)**	**C**_**trough**_ **(mg/L), median (range)**	**C**_**trough**_**/dose (mg/L)/kg/day), median (range)**
**Total group**	39*		
CYP2C19*1*1	16 (41)	1.32 (0.10–6.75)	0.104 (0.005–0.430)
CYP2C19*1*2	7 (18)	1.42 (0.22–4.36)	0.108 (0.233–0.276)
CYP2C19*1*17	11 (28)	0.23 (0.10–1.31)	0.024 (0.024–0.159)
CYP2C19*17*17	5 (13)	0.41 (0.15–1.53)	0.071 (0.008–0.102)
**Patients < 12 years of age**	16		
CYP2C19*1*1	6 (38)	1.48 (0.10–3.85)	0.077 (0.005–0.250)
CYP2C19*1*2	2 (12)	3.29 (2.21–4.36)	0.242 (0.207–0.276)
CYP2C19*1*17	5 (31)	0.19 (0.1–1.3)	0.010 (0.010–0.080)
CYP2C19*17*17	3 (19)	0.33 (0.15–1.53)	0.017 (0.017–0.089)
**Patients ≥ 12 years of age**	23		
CYP2C19*1*1	10 (43)	1.16 (0.1–6.75)	0.123 (0.013–0.430)
CYP2C19*1*2	5 (22)	1.36 (0.22–2.02)	0.096 (0.033–0.228)
CYP2C19*1*17	6 (26)	0.35 (0.15–1,07)	0.064 (0.009–0.159)
CYP2C19*17*17	2 (9)	0.63 (0.41–0.63	0.086 (0.070–0.102)

^*^Of the forty-four blood samples collected from pediatric patients, thirty-nine were tested for the CYP2C19 phenotype, as five of the blood samples were hemolyzed

**Table 5:** CYP2C19 metabolizers influencing probability of the therapeutic range of VCZ

CYP2C19 phenotype	VCZ C_trough_ N (%)		
	Subtherapeutic (< 1.0 mg/L)	Therapeutic ( 1.0–5.5 mg/L)	Supratherapeutic (> 5.5 mg/L)
NN	5 (31)	10 (63)	1 (6)
IM	2 (29)	5 (71)	0 (0)
RM	7 (64)	4 (36)	0 (0)
UM	4 (80)	1 (20)	0 (0)

*NM*-normal metabolizers, *IM*-intermediate metabolizers, *RM*-rapid metabolizers and *UM*-ultrarapid metabolizers

## Discussion

Due to the high inter-individual variability in plasma concentration and clinical response, there is a growing interest in personalizing VCZ therapeutic strategies for each patient. We studied VCZ TDM in 44 immunocompromised children, and in over 50% of cases, subtherapeutic plasma C_trough_ of VCZ was observed. These results are comparable with data from previous studies performed by Liu et al., Lampers et al., Duehlmeyer et al., and Tucker et al. [44–47]. Brüggeman et al. showed that 44% of 18 children, Neely et al. 34% of 46 patients, and Spriet et al. 31% of 16 children > 2 years of age receiving VCZ of the recommended dose, C_trough_ not achieved the target concentration of 1 mg/L [17, 48, 49]. Our study showed high inter-individual variability of VCZ C_trough_, ranging from 0.1 to 6.8 mg/L. No predictable relationship between dose and VCZ C_trough_ was observed in analyzed patients (P = 0.267), due to the nonlinear pharmacokinetics of VCZ [50]. Other studies reported the same results in terms of plasma variability [7, 44]. A very low correlation between VCZ C_trough_ and the dose was found by Spriet et al. [49, 51]. Similarly, as previously reported studies [7, 30, 44, 49], no significant differences in the number of VCZ C_trough_ within the therapeutic range between patients < 12 years and ≥ 12 years (P = 0.192) was observed (Fig. 2).However, the correlation between the age and VCZ C_trough_ were identified by Allegra et al., Mori et al., Kato et al. and Hicks et al. [33, 51–53]. Despite the lack of correlation in older children, a positive relationship between age and C_trough_ was observed in younger children below 2 years old in the study performed by Liu et al. [45]. However, due to the absence of patients of this age in the studied group, we could not confirm it. The influence of the route of administration on C_trough_ concentration was also investigated. No significant difference was found in the median VCZ C_trough_ between children with oral and intravenous administration in both groups (P = 0.903 and P = 0.71). Similar results were obtained by Soler-Palacin et al. and Barteling et al. [7, 12]. In contrast, Kato et al. reported that oral administration was associated with significantly lower VCZ C_trough_ than that associated with intravenous administration in children aged ≤ 12 years [52].

Additionally, in the current study, similarly to previous reports [36, 45, 50, 54], no significant effect of sex on VCZ C_trough_ was observed. The obtained results were contrary to those reported by Allegra et al. [33]. The authors concluded that sex significantly influenced VCZ level; with a higher median drug concentration for the male group (P < 0.001). This is a single study in that a broad group of patients was studied (237 children enrolled).

There is an agreement that younger children require higher maintenance doses to attain therapeutic C_trough_. However, there are no guidelines for dose adjustment of VCZ in children, so it is often at the discretion of the clinicians. Early published data suggest that the recommended dosages for pediatric patients were inadequate to achieve C_trough_ greater than 1 mg/L, especially for younger children under the age of 12 [47, 49, 55, 56]. In the study performed by Spriet et al. median dose of 6.95 mg/kg, every 12 h was examined [49]. The authors confirmed that VCZ concentrations varied widely in children younger than 12 years of age ranging from 0.09 to 4.90 mg/L while in patients > 12 years old ranging from 0.11 to 1.71 mg/L. However, we did not perform VCZ dosing optimization based on TDM.

We found a consistent association between VCZ C_trough_ and treatment outcome. The median VCZ C_trough_ was significantly lower in patients who failed treatment 0.19 mg/L (range: 0.1–1.68 mg/L) than in patients treated successfully 1.31 mg/L (range: 0.10–6.75 mg/L) (P < 0.05). These findings are similar to those reported by Kang et al. [20]. That supports the need for VCZ TDM and demonstrates its significant correlation with improved clinical outcomes.

Another critical factor affecting VCZ C_trough_ is the polymorphism of CYP2C19. This pharmacogenetic study was performed and as a result, the CYP2C19*2 and the CYP2C19*17 alleles were presented. In our study, the diplotype *1*1 was present at a relatively high frequency, and it was in line with the data previously published [43]. Clinical Pharmacogenetics Implementation Consortium (CPIC) reported CYP2C19 NMs in approximately 35–50% of individuals. The proportion of CYP2C19 PMS varies with race-ethnicity. It has been shown that PMs phenotype is present in only about 3–7% of Caucasians. No CYP2C19*2*2 alleles were found in our study. The study of the clinical impact of CYP2C19 genetic variants on VCZ pharmacokinetics in children is still controversial due to limited data availability. It has been proposed that VCZ exposure in immunocompromised pediatric patients could not be predicted based on CYP2C19 genotype [36, 57]. In our study, VCZ C_trough_ was higher in the CYP2C19 NM and IM groups compared with the RM and UM groups (Fig. 3), and it was contrary to the results observed by Driscoll et al. Additionally, the median of VCZ C_trough_ obtained from patients who carried CYP2C19*17 allele was less than 0.5 mg/L, which may be a risk factor for increased mortality [6]. Our results partially corroborate previous findings in pediatric patients. Hick et al. found that CYP2C19 genotypes were associated with VCZ in children. VCZ dose normalized plasma C_trough_ were significantly lower in UMs phenotype patients (P = 0.04) and significantly higher in PMs phenotype patients (P = 0.04); than those with NMs phenotype patients. It was shown that children with UMs phenotype never achieved VCZ target range at any dose. The authors concluded that CYP2C19*17 homozygous pediatric patients would require higher doses to reach the VCZ target concentrations [35]. Another analysis performed by Narita et al. showed that C_trough_ of VCZ were significantly higher (P = 0.004) in the PMs and IMs metabolizer groups, compared with the NMs metabolizers and UMs groups [30]. Teusink et al. analyzed 20 children with a median age of 10.9 years undergoing HSCT who received CYP2C19 genotype-directed dosing [38]. Results showed a significant difference in doses and time required to achieve C_trough_ of VCZ for different CYP2C19 genotypes. The algorithm according to the individual CYP2C19 genotype was performed. The authors found that in the follow-up study, no patients had C_trough_ lower than < 1 mg/L and higher than > 5.5 mg/L. It showed a statistically significant (P < 0.001) decrease in time to reach the VCZ target range of 1–5.5 mg/L from 29 to 6.5 days when genotype-guided dosing was used. The strategy consisted of a combination of genotyping of CYP2C19 and routine TDM in children was also evaluated by Garcia-Garcia et al. [58]. The authors showed that the dose modification based on preemptive genotyping allows achieved target concentration in 90% of CYP2C19 NM and IM metabolizers, and 100% of the RM and UM metabolizers. Although we did not perform a pharmacokinetics model stratified by CYP2C19 diplotype in our study, we are in agreement that dose modifications based on pharmacogenetic information could be a useful tool for VCZ therapy individualization.

The activity of ALT and AST in serum, and serum ALB level had no significant effect on the pharmacokinetics of VCZ in this study. These results can be attributed to the fact that for the majority of patients enrolled in the study, these parameters were within the reference ranges. Only one study of the pediatric population considered liver dysfunction as a factor involved in the variation of plasma concentration of VCZ between individuals [59]. Zeng et al. found a significant correlation between ALB and VCZ concentration (P < 0.001) while a statistical effect was not observed for the ALT and AST enzymes. This finding confirms other observations performed by Zeng et al. and Lin et al. in adult patients [59, 60]. The authors did not find any correlations with liver function indicators and the achieved VCZ C_trough_. Nevertheless, some studies reported that decreased liver function resulted in a significant reduction in VCZ metabolism so it is essential to monitor VCZ concentration closely in patients with hepatic dysfunction [61, 62].

Due to the fact that VCZ is an inhibitor and substrate of CYP2C19, 2C9, and 3A4, drug-drug interactions with agents metabolized by these pathways are common. Coadministration of these drugs could be a rationale for VCZ TDM**.** Coadministration of VCZ with CYP2C19 substrates such as PPIs may increase VCZ plasma concentrations due to competitive inhibition, but the degree of affect depends on the type of PPIs [60]. In our study, we found no significant differences in VCZ C_trough_ between groups treated with PPIs when compared with patients without PPIs treatment. Similar findings were also observed in a study by Spriet et al. [49]. The authors did not identify PPIs as a significant covariate influencing VCZ metabolism. In contrast, Hu et al., in a comparably large group, reported that VCZ coadministered with any PPI resulted in significantly higher C_trough_ than VCZ administration alone (P = 0.028) [50]. In a study by Liu et al. it was found that omeprazole significantly increased the C_trough_ of VCZ in pediatric patients (P = 0.032) [60]. Very limited data on the effect of CsA on VCZ pharmacokinetics are available. Zeng et al. reported no significant differences in VCZ C_trough_ between the patients coadministered with or without CsA [63]. Similar observations were presented in the study conducted by Heshemizadeh et a. in liver transplantation patients [64]. However, Lelievre et al. evaluated that CsA coadministration was significantly associated with an increase in VCZ concentration in plasma and cerebrospinal fluid in a study performed in a rat model [65]. These observations were confirmed by a recent study elaborated by Chen et al. in immunocompromised children [36]. The authors postulated that the respective involvement of CYP2C19 and CYP3A4 cytochromes depends on VCZ level. In the case of low concentration of VCZ, CYP2C19 was mainly responsible for the metabolism, whereas enzyme CYP3A4 was mainly responsible for the metabolism in the case of high concentration of VCZ. VCZ disposition might be modified by CYP3A4 catalytic site, at a high level of VCZ. In connection with these reports, clinicians should pay particular attention to the concomitant use of these drugs.

This study has several limitations. Firstly, this study was performed in a single highly specialized center with a limited number of patients. Secondly, those patients who did not achieve therapeutic range in the first measure of VCZ C_trough_ after dose modifications were not subjected to a subsequent TDM analysis. Therefore, we do not have results indicating that TDM-based dosing adjustment reduced the proportion of patients reaching subtherapeutic C_trough_ of VCZ. Furthermore, the potential phenoconversion effect was not studied. Concomitant drugs that inhibit cytochrome P450 enzyme activity or induce their expression, as well as disease-related factors, can cause phenoconversion effects. For CYP2D6 tool was established to integrate standardized assessments of phenoconversion in clinical practice. To our knowledge, data on the relevance of phenoconversion for CYP2C19 still needs to be included. In a study performed by de Jong et al. [66], the authors described that for omeprazole, phenoconversion into IM or PM phenotypes was in only 10% of the donors (3/30). It was in contrast to a clinical study performed by Klieber et al. [67], where 96%of patients converted to a PM phenotype after treatment with omeprazole or esomeprazole. In our case, omeprazole (PPI) was potential pertubators of CYP enzymes. However, a few patients (9/44) were coadministered with VCZ and PPI. Additionally, no PM phenotype was observed. Nevertheless, our studies indicate that the CYP2C19 polymorphism is one of the factors influencing the inter-individual variability of VCZ plasma concentration and indicates the value of CYP2C19 genotyping in selecting appropriate VCZ dosage for pediatric patients. Further prospective studies are still needed to be performed in the pediatric population to validate our findings.

## Conclusion

In conclusion, the C_trough_ of VCZ is characterized by inter-individual variability and a low rate of patients reaching the therapeutic range. Taking into account these results, we can see that there is a need to improve VCZ dose predictions and that pharmacogenetics represents a helpful tool to optimize therapy. In our opinion, a combination of both strategies genotyping, routine, and repeated TDM, is extremely necessary in order to ensure the safety and effectiveness of treatment.

## Data Availability

The datasets generated during and/or analysed during the current study are available from the corresponding author on reasonable request.
